# Methylthioadenosine (MTA) boosts cell‐specific productivities of Chinese hamster ovary cultures: dosage effects on proliferation, cell cycle and gene expression

**DOI:** 10.1002/2211-5463.13019

**Published:** 2020-11-11

**Authors:** Natascha Verhagen, Julia Zieringer, Ralf Takors

**Affiliations:** ^1^ Institute of Biochemical Engineering University of Stuttgart Stuttgart Germany

**Keywords:** cell cycle arrest, cell‐specific productivity, Chinese hamster ovary cell, medium optimization, methylthioadenosine, transcriptome analysis

## Abstract

A major goal for process and cell engineering in the biopharmaceutical industry is enhancing production through increasing volumetric and cell‐specific productivities (CSP). Here, we present 5′‐deoxy‐5′‐(methylthio)adenosine (MTA), the degradation product of S‐(5′‐adenosyl)‐L‐methionine (SAM), as a highly attractive native additive which can boost CSP by 79% when added to exponentially growing cells at a concentration of 250–300 μm. Notably, cell viability and cell size remain higher than in non‐treated cultures. In addition, cell cycle arrests first in S‐, then in G2‐phase before levelling out compared to non‐treated cultivations. Intensive differential gene analysis reveals that expression of genes for cytoskeleton mediated proteins and vesicle transport is amplified by treatment. Furthermore, the interaction of MTA with cell proliferation additionally stimulated recombinant protein formation. The results may serve as a promising starting point for further developments in process and cell engineering to boost productivity.

## Graphical abstract

The impact of addition of methylthioadenosine (MTA), a stimulator of cell‐specific productivity (CSP) in Chinese hamster ovary (CHO) cells, is concentration and time dependent. Moreover, MTA affects cell cycle, cell size, and viability. Transcript analysis of post‐MTA addition revealed interesting targets for cell line and process engineering.

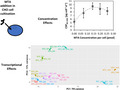

**Abbreviations**
CHO: Chinese hamster ovary
CSP: cell‐specific productivity
DEG: differential expressed gene
MTA: 5’‐deoxy‐5’‐(methylthio)adenosine
PC: principal component
REF: reference
SAM: S‐(5’‐adenosyl)‐L‐methionine
VCD: viable cell density

Biopharmaceutical markets are dominated by therapeutic proteins, particularly monoclonal antibodies (mAB) which are predominantly produced by CHO cells [1]. In the last decades, significant increase of maximum viable cell density (VCD) improved volumetric productivity and reached titers up to 5–8 g·L^−1^ in fed‐batch processes [2, 3, 4]. Process intensifications are performed to raise production performance. As a prerequisite, increasing CSPs are needed for the next step of process development [5, 6].

5′‐Deoxy‐5′‐(methylthio)adenosine (MTA) consists of L‐methionine (L‐met) and adenosine triphosphate (ATP) and is a naturally occurring molecule in mammalian tissues [7, 8]. It is produced from S‐(5′‐adenosyl)‐L‐methionine (SAM) in the polyamine synthesis [7] in cells. Production of spermidine and spermine needs the decarboxylation of SAM to MTA that is rapidly metabolized by 5'‐methylthioadenosine phosphorylase to adenine and S‐methyl‐5‐thio‐D‐ribose 1‐phosphate and finally to L‐met. The adenine can be used to replenish adenosine monophosphate (AMP), adenosine diphosphate (ADP) and ATP pools. Final recovery of SAM from ATP and L‐met closes the SAM cycle [7, 9]. Rapid degradation of MTA is crucial because it inhibits spermine synthase, spermidine synthase and ornithine decarboxylase [8, 10].

MTA inhibited cell proliferation in hepatocytes, leukemia cells, fibroblasts and lymphoma cells [11, 12, 13, 14] that is mainly the consequence of its polyamine synthesis inhibition [15]. A reduction of polyamine intermediates arrested CHO cells in their S‐phase [16]. Furthermore, MTA addition inhibited DNA synthesis in hepatic cells [10] but it remained unclear whether MTA or a downstream metabolite is the effector [17].

Beside its interaction with polyamine synthesis, MTA demonstrated importance for expression control of genes, cell proliferation inhibition, lymphocyte activation, tumor development and invasiveness, and the regulation of apoptosis [7, 9, 10, 11, 12, 18]. MTA addition induced apoptosis in hepatocarcinoma cells, whereas hepatocytes remained viable and were protected against programmed cell death [11]. Additionally, MTA demonstrated beneficial effects in immune response [19].

Several groups [13, 14] observed the inhibition of growth factor‐induced protein tyrosine phosphorylation and the increase of intracellular cyclic AMP (cAMP) levels through the inhibition of cAMP‐phosphodiesterase by MTA pointing out the interaction with signaling pathways. Furthermore, increased MTA levels inhibited arginine methylation of the STAT1 transcription factor, finally impairing gene transcription [20].

Due to the relation to the SAM cycle, MTA revealed capabilities to inhibit protein methylation pinpointing to its role as post‐translational modifier and accordingly as a regulator of cellular signaling and gene expression [12, 18, 20]. Evidences are given by its direct interaction with methyltransferases and via the indirect inactivation of S‐(5’adenosyl)‐L‐homocysteine hydrolase [7].

Single MTA addition to the medium increases CSP in CHO cells. Furthermore, cells demonstrated cell cycle arrest and increased cell size [21]. Growth arrest induction is a common strategy to increase CSP [22]. Protein production was increased by effector‐induced cell cycle arrest in G1‐ and S‐phase [23, 24]. However, cell size controls transitions between cell cycle phases which underlines its importance for proper cell cycling and proliferation [25, 26, 27] that correlated with protein production in different cell lines [28]. The complex interactions between cell size, cell growth, and protein production are not fully elucidated, yet. Additionally, genes involved in post‐translational steps, secretion and cytoskeleton were reported to enhance CSP [29, 30, 31].

Strategies to induce growth arrest for enhancing protein production comprise (a) hypothermia and (b) the addition of effector molecules, e.g. to increase hyperosmolality. Regarding (a), the mechanism of hypothermia is not understood but certainly linked to G1‐phase arrest [32] and accompanied by an increased cell size [33, 34]. With respect to (b) additives were investigated to modulate cell growth, product stabilization, and to reduce chemical modifications. Examples are sodium butyrate [35], zinc [36], valeric acid [37], glycine betaine [38], valproic acid [39] and sodium chloride [40] among others.

Own studies have already revealed that MTA addition diminished growth, increased CSP, altered cell cycle phases and cell size [21]. Consequently, MTA should be considered as a multi‐layer regulator of cell growth, cell cycle, and protein formation that is a highly promising additive for boosting CSP. Accordingly, we conducted experiments with anti‐IL‐8‐producing CHO cells analyzing different levels and intervals of MTA addition and the effect of MTA on transcriptomic level.

## Materials and methods

### Different MTA concentrations and addition time points: Seed train, shake flask cultivation and MTA addition

MTA was a product of Sigma‐Aldrich (Steinheim, Germany). The anti‐IL‐8‐producing CHO DP‐12 cell line (ATCC^®^ CRL 12445™) adapted to suspension was grown in chemically defined TC‐42 medium (Xell AG, Bielefeld, Germany) supplemented with 4 mm L‐glutamine (Carl Roth GmbH & Co. KG, Karlsruhe, Germany) and 200 nm methotrexate (Sigma‐Aldrich). Seed train and experiments were performed in pre‐sterilized disposable shake flasks (Corning Inc., US) in a humidified and incubated rotary shaker (Infors HT Minitron, Infors GmbH, Germany) at 37 °C, 150 rpm with 50 mm displacement and 5% CO_2_. In the experiment with different concentrations, MTA was introduced after 48 h of cultivation in different concentrations (125, 250, 350 and 450 µm). In reference (REF) cell cultures, sterilized water was used to mimic the additional liquid volume in experimental cultures (volume corresponding to the 450 µm addition). In the experiment with different addition time points, MTA was introduced after 48, 84 and 108 h of cultivation in a concentration of 150 pmol·cell^−1^. At every addition time point, all other settings received sterilized water to mimic the additional liquid volume in experimental cultures. Cultivation was performed with biological duplicates.

### Extracellular and cell cycle analysis

Samples were taken at least once a day. Viable cell density (VCD), viability and average cell size were determined using trypan blue staining and a Cedex XS cell counter (Innovatis AG, Bielefeld, Germany). The extracellular concentrations of D‐glucose (D‐Glc) and L‐lactate (L‐Lac) were determined using a LaboTRACE automatic analyzer (Trace Analytics GmbH, Braunschweig, Germany). The concentration of secreted antibody was determined with an enzyme‐linked immunosorbent assay (ELISA) [41]. All sampling and measurement procedures were performed with three technical replicates. The determination of cell cycle distribution was performed as described before [21]. All sampling and measurement procedures were performed with two technical replicates.

### Transcriptome analysis

Experimental equipment and settings were used as described above (MTA at 48 h: 250 µm) in biological triplicates. The isolated RNA was processed by c.ATG. Analysis of raw data was performed on the Galaxy‐Server [42], and data were analyzed using the free statistical computing environment R.

#### Experiment and sampling for transcriptome analysis and ribonucleic acid (RNA) sequence analysis

Experimental equipment and settings were described in the manuscript. Sampling for transcriptome analysis occurred on 48‐h, 60‐h, 72‐h, 84‐h, 96‐h and 144‐h cultivation time and followed an adapted protocol [40]. A total number of 2 × 10^6^ cells were harvested and centrifuged, and supernatant was discarded. Cells were resuspended in RNAprotect Cell Reagent (Qiagen, Hilden, Germany), quickly frozen in liquid nitrogen and stored at −70 °C. The RNA was isolated with the RNeasy Kit (Qiagen) and QiaShredder (Qiagen). An extra procedure of clean‐up to get rid of DNA (Turbo DNaseTM and Turbo DNaseTM Buffer, Ambion (Life Technologies, Carlsbad, CA, USA)) and increase the RNA concentration (RNA Clean & ConcentratorTM, Zymo Research, Irvine, CA, USA) was added. The kits were used as indicated by the manuals. Sequencing of the transcriptome was performed by c.ATG (Tübingen, Germany). Preparation of high‐quality mRNA‐Seq data was performed using the Illumina TruSeq RNA Sample Preparation Kit. Quality was assessed by an Agilent Fragment Analyzer. Samples with high RNA integrity number (RIN > 8) were selected for library construction using the NEBNext Ultra II Directional RNA Library Prep Kit. Libraries were sequenced as paired‐end (50 bp read length) at a depth of 30–40 million reads each.

#### Read mapping and gene counting

Read mapping and gene counting was performed on the Galaxy‐Server. Sequencing statistics including the quality per base and adapter content assessment of resulting transcriptome sequencing data were checked by FastQC reports. Genes were aligned to the CHO‐K1 reference genome (RefSeq: GCF_000223135.1) (downloaded from http://www.chogenome.org/, 07/08/2019) using the RNA sequencing aligner bowtie2 v. 2.3.2.2 [43]. On average, the mapping of the reads covers 94.3%. Aligned reads were counted for each gene based on the corresponding annotation available from the CHOgenome wegpage for the chosen reference sequence applying htseq‐count v. 0.6.1 [44] in the union mode. On average, 71.0% of the sequenced reads could be assigned uniquely to annotated genes. Sequencing depth was around 33 million reads per sample on average.

#### Transcriptome data analysis

Differential gene expression analysis was performed with the R‐package deseq2 v. 1.26.0 [45] available from Bioconductor [46] (http://www.bioconductor.org). Prior to statistical analysis, a non‐specific filter was applied to remove low coverage genes with less than one count per million (33 reads on average) in two out of three replicates per condition. Samples were grouped by replicates, and an experimental design was chosen that used sample time and treatment (CPC, REF, MTA) as a combined environmental factor. To normalize the read counts for comparison purposes on sequencing depth and RNA composition, DESeq2 uses the median of ratios method to derive a scaling factor. Dividing the original read counts by the scaling factor normalized count values are generated. To model count‐based expression data, DESeq2 uses a negative binomial model as a distribution assumption and fits the expression data for each gene to a generalized linear model (GLM). No outliers were observed in the three biological replicates using Pearson correlation. Resulting *P*‐values were adjusted for multiple testing according to [47] to control the false discovery rate (FDR). Genes were identified as significantly differentially expressed by applying FDR adjusted *P*‐values < 0.05 and a log2‐fold‐change ≥ |1|. A principal component analysis was used to display the sample to sample distances calculated within the DESeq2 package using the function plotPCA.san available on Github (https://gist.github.com/sansense/3399064897f1252d31b23ea5178c033c). Raw counts and processed data can be found in the supplementary information. Data analysis was performed using the free statistical computing environment r v. 3.6.2.

## Results

### The effect of MTA addition depends on its concentration

**Fig. 1:**
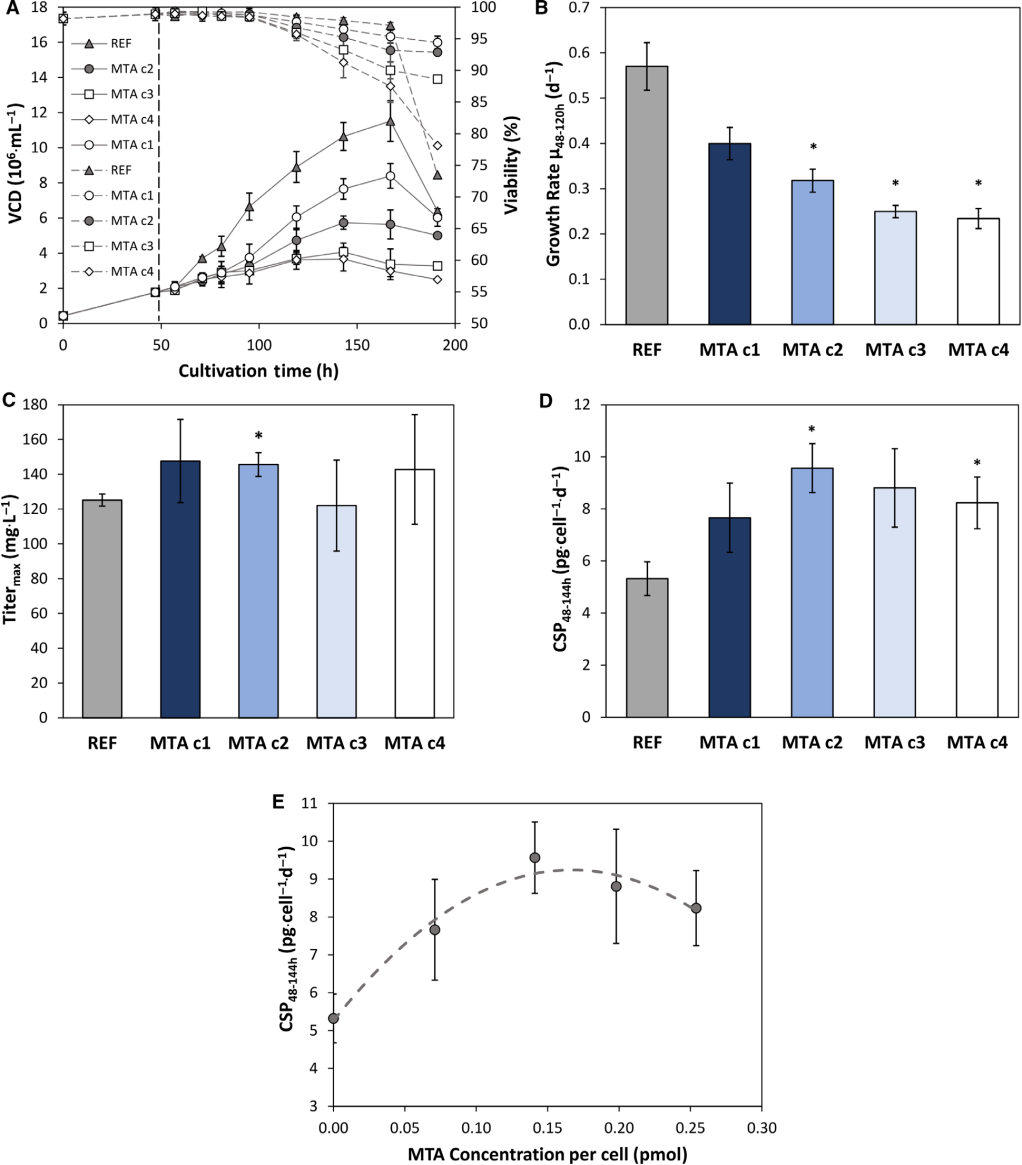
(A) VCD [10^6^ cells·mL^−1^] and viability [%] of MTA supplemented cells and reference (REF▲). MTA was added at 48 h in different concentrations: c1 150 µm(○), c2 250 µm(●), c3 350 µm(□), c4 450 µm(◊). (B) Growth rate per day [d^−1^] regarding the time interval 48–120 h. (C) Maximum antibody titer [mg·L^−1^]. (D) CSP [pg·cell^−1^·d^−1^] regarding the time interval 48–144 h. (E) CSP [pg·cell^−1^·d^−1^] between 48–144 h plotted against the MTA amount per cell [pmol] at 48 h. Error bars show standard deviations of biological duplicates. Significance (to REF) was tested with a t‐test; * < 0.05.

MTA was added in different concentrations (c1: 150 µm, c2: 250 µm, c3: 350 µm, c4: 450 µm) to the cells after 48‐h cultivation time. All MTA treated cultures showed reduced VCD and growth rate (regarding 48–120 h) dependent on the concentration (Fig. 1). Higher MTA amounts reduced VCD and growth rate stronger than low additions. The lowest concentration c1 led to 30% reduction of growth rate whereas the two highest concentrations c3 and c4 halved it. c2 reduced growth rate by 44%. However, maximum inhibition trends were observed for c3 and c4: The reduction of growth rate plateaued. Addition of MTA with c1, c2 and c3 demonstrated a higher viability in the last cultivation phase compared to REF. By trend, analysis of maximum product titers unraveled slightly elevated amounts of antibodies for all MTA additions except c3 (Fig. 1). Calculating cell‐specific productivities revealed boosted CSPs for all MTA additions between 48–144 h. Concentration c2 showed the best performance (+79.7%) and c1 the lowest (+43.9%). Fitting the CSPs to a 2^nd^ order polynomial function reveals optimum MTA addition of 0.167 pmol_MTA_·cell^−1^ at 48 h (Fig. 1). The equivalent medium concentration of 295.59 µm is close to the tested level of c2 with the highest CSP in the experimental series.

**Fig. 2:**
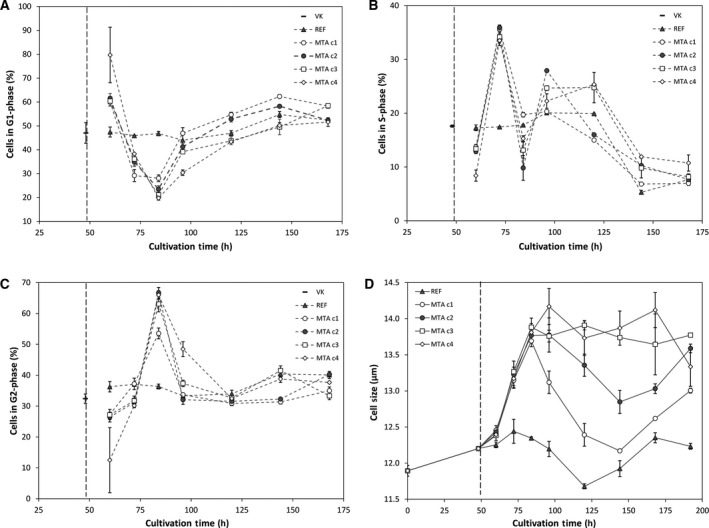
Cell cycle phase distribution (A–C) [%] and average cell size (D) [µm] of MTA supplemented cells, reference (REF▲) and common preculture (—). MTA was added at 48 h in different concentrations: c1 150 µm(○) c2 250 µm(●), c3 350 µm(□), c4 450 µm(◊). Error bars show standard deviations of biological duplicates.

Cell cycle phase distribution revealed the concentration‐dependent effect of MTA (Fig. 2). A common preculture split right before MTA (48 h) served as a starting point. Half a day after MTA addition, cells accumulated in G1‐phase. 12 h later, i.e. one day after addition, the number of cells in S‐phase increased for the sake of those in G1‐phase. At 84 h (36 h after addition) cells in G2‐phase dominated and the number of cells in G1‐phase kept dropping. Two days after addition the ratios started to normalize. Cultures with c3 and c4 approached REF conditions whereas c1 and c2 kept an elevated fraction of cells in G1‐phase. The different MTA concentrations caused diverse effects on cell size. In general, cell size was smallest in REF and largest after c3 and c4 addition. By trend, all MTA treated cells kept enlarged cell size on different levels compared to REF.

### The effect of MTA is time‐dependent

**Fig. 3:**
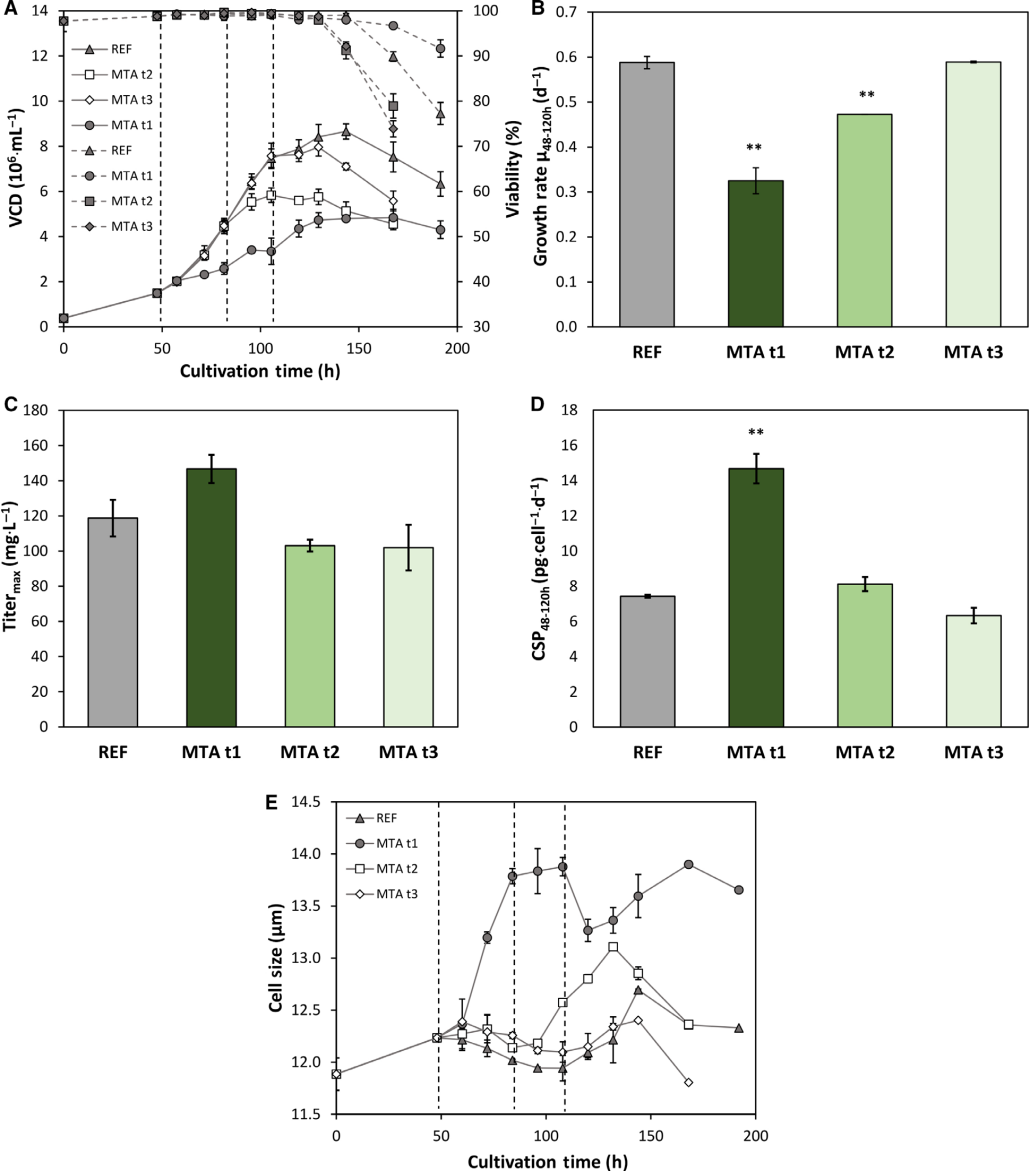
(A) VCD [10^6^ cells·mL^−1^] and viability [%] of MTA supplemented cells and reference (REF▲). MTA was added with 0.167 pmol_MTA_per cell at 48, 84 or 108 h: MTA t1 48 h (●), MTA t2 84 h (□), MTA t3 108 h (◊). (B) Growth rate per hour [d^−1^] regarding the time interval 48–120 h. (C) Maximum antibody titer [mg L^−1^]. (D) CSP [pg·cell^−1^·d^−1^] regarding the time interval 48–120 h. (E) Average cell size [µm]. Error bars show standard deviations of biological duplicates. Significance (to REF) was tested with a*t*‐test; ** < 0.01.

In another experimental series, cells received 0.167 pmol_MTA_·cell^−1^ after 48, 84 and 108 h (Fig. 3). The rise of VCD slowed down after each MTA addition. Growth reduction was more pronounced the earlier MTA was added with the 48‐h‐shot showing the slowest post‐MTA growth rate. However, the viability of the treated cells remained even higher than the performance of REF. Regarding growth rate the 48‐h‐addition caused 44.7% reduction whereas the 84‐h‐addition only reduced growth by 19.6%. Late addition (108 h) did not cause any growth difference compared to REF. Maximum antibody titers [mg·L^−1^] did not increase after MTA additions at 84 and 108 h (Fig. 3) but rose after 48 h. The trend is even more pronounced with respect to CSPs. The 48‐h‐supplementation almost doubled CSP (+97.4%) compared to REF whereas later MTA treatments showed no effects. By analogy, cell size raises the most when MTA was added at 48 h.

**Fig. 4:**
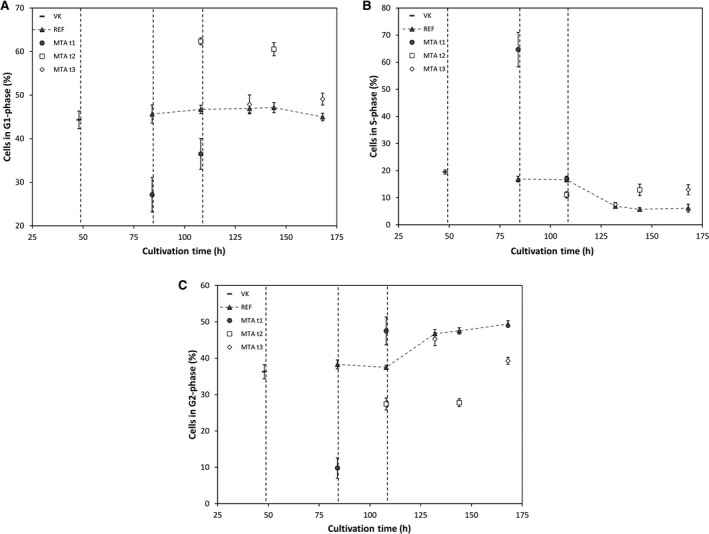
Cell cycle phase distribution (A–C) [%] of MTA supplemented cells, reference (REF▲) and common preculture (—). MTA was added with 0.167 pmol_MTA_per cell at 48, 84 or 108 h: MTA t1 48 h (●), MTA t2 84 h (□), MTA t3 108 h (◊). Error bars show standard deviations of biological duplicates.

Cell cycle phase distributions and cell sizes are displayed in Fig. 4. Again, highest impact was found for 48‐h‐cultures whereas later MTA addition did not reveal strong differences compared to REF. Early supplementation caused increasing cell fractions in S‐phase and decreasing percentages in G1‐ and G2‐phase 36 h after addition. 60 h after addition, the partition of cells in G2‐phase increased and there were still less cells in G1‐phase.

### Monitoring transcriptional responses after MTA addition

**Fig. 5:**
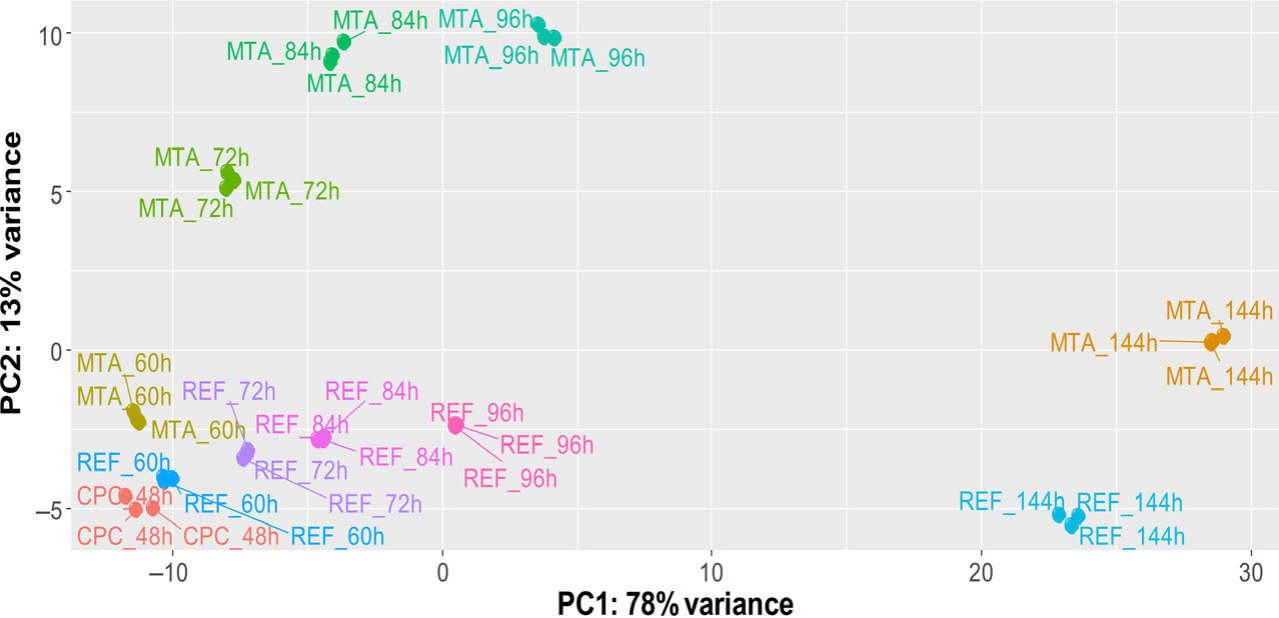
Principal component (PC) analysis of the transcriptome samples taken in the experiment. The two main principal components are cultivation time (PC1) and condition (MTA treatment, PC2). Samples were taken in the common preculture (CPC) at 48‐h cultivation time and after MTA addition (final concentration: 250 µm) at the cultivation time points 60, 72, 84, 96 and 144 h. Reference (REF) cultures received the equal volume of water to avoid dilution effects.

**Fig. 6:**
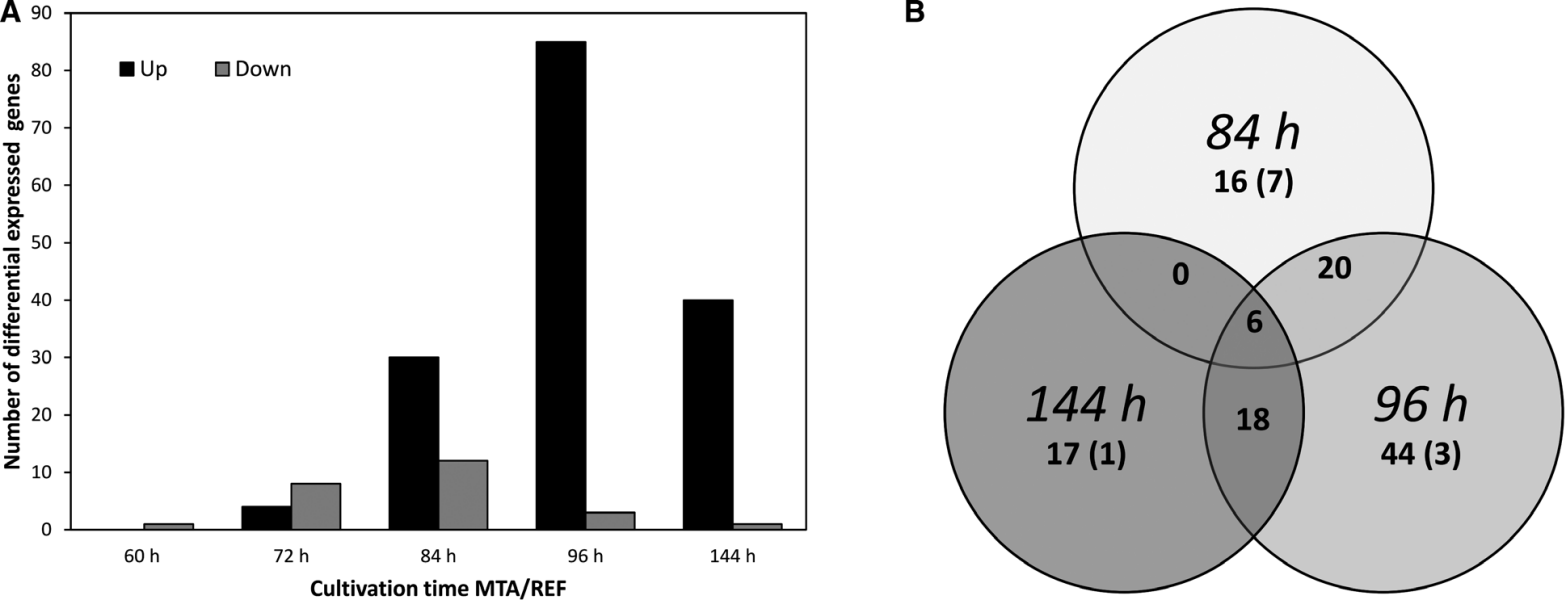
(A) Analysis of differential expressed genes (DEGs) (log_2_‐fold‐change ≥|1| and*P*‐value ≤ 0.05) throughout the experiment. MTA supplemented cells (MTA) were compared to REF. Grey bars indicate downregulated and black bars show upregulated genes at different sampling time points. (B) Venn diagram shows overlap of DEGs at 84 h–96 h–144 h. Numbers display all DEGs with the number of downregulated genes in brackets.

The impact of MTA on the transcriptome was evaluated via differential gene expression (DEG) analysis based on RNA sequencing. mRNAs of biological triplicates supplemented with MTA were compared at different time points (60, 72, 84, 96, 144 h) to REF. Around 91% of total variance is covered by two principal components (PC) clearly grouping biological triplicates of equal sampling points. Apparently, cultivation time is represented by PC1 and MTA addition by PC2 (Fig. 5). In total, 122 DEGs were identified according to the constraints log_2_‐fold‐change ≥|1| and adjusted *P*‐value ≤ 0.05. Downregulation of genes occurred mostly 12–36 h after addition (60 h: 1, 72 h: 8, 84 h: 12, 96 h: 3, 144 h: 1) (Fig. 6). Later, i.e. 84 h process time, upregulation of genes dominated DEGs by far (60 h: 0, 72 h: 4, 84 h: 30, 96 h: 85, 144 h: 40). The Venn diagram comprising DEGS at 84 h–96 h–144 h reveals 6 commonly upregulated genes compared to REF. All DEGs that were significantly up‐ or downregulated at more than one sampling time point are listed in Table 1.

**Table 1:** Selection of differential expressed genes. Downregulated genes are highlighted in grey.

Cultivation time	Gene name	Encoded protein
84 h–96 h–144 h	*Plau*	Urokinase‐type plasminogen activator
	*Aqp1*	Aquaporin‐1
	*Lcp1*	Plastin‐2
	*St14*	Suppressor of tumorigenicity 14 protein homolog
72 h–84 h	*Apc2*	Adenomatous polyposis coli protein 2
	*Il11*	Interleukin‐11
84 h–96 h	*Rassf6*	Ras association domain‐containing protein 6
	*Abhd6*	monoacylglycerol lipase ABHD6
	*Hr*	Lysine‐specific demethylase hairless
	*Dnase1l3*	Deoxyribonuclease gamma
	*Il17f*	Interleukin‐17F
	*Lpin3*	Phosphatidate phosphatase LPIN3
	*Arnt2*	Aryl hydrocarbon receptor nuclear translocator 2
	*Sh2d1b*	SH2 domain‐containing protein 1B
	*Fam110c*	Protein FAM110C
	*Cd53*	Leukocyte surface antigen CD53
	*Oasl*	2'‐5'‐oligoadenylate synthase‐like protein 2
	*Egfr*	Epidermal growth factor receptor
	*St8sia6*	Alpha‐2,8‐sialyltransferase 8F
96 h–144 h	*Add2*	beta‐adducin
	*Loxl2*	Lysyl oxidase homolog 2
	*Il7r*	Interleukin‐7 receptor subunit alpha
	*Tmprss11f*	Transmembrane protease serine 11F
	*Adgrd1*	Adhesion G‐protein coupled receptor D1
	*Sema4d*	Semaphorin‐4D
	*Akr1d1*	Aldo‐keto reductase family 1 member D1
	*Mpeg1*	Macrophage‐expressed gene 1 protein
	*Pglyrp2*	N‐acetylmuramoyl‐L‐alanine amidase

Four genes of the six DEGs observed at 84 h–96 h–144 h are annotated: *Aqp1*, *Lcp1*, *Plau* and *St14* whereas two are unknown loci. Aquaporin 1 (*Aqp1*) is a commonly amplified water channel. *Lcp1* codes for plastin, an actin‐binding cellular component. Plasminogen activator (*Plau)* and matriptase (*St14*) are serine proteases.

A lot of genes are differentially expressed at two time points after MTA addition. 12 and 24 h after MTA addition (72 h–84 h) two annotated genes are differentially expressed: Adenomatous polyposis coli protein 2 (*APC2*), a gene transcription regulator is downregulated whereas *Il11* (interleukin‐11) is upregulated. 24 and 36 h after MTA addition (84 h–96 h) 13 additional upregulated DEGs were observed: (a) the small G‐protein (ras) associated domain‐containing protein 6 (*Rassf6*) which is associated with cellular apoptosis, (b) the epidermal growth factor receptor (*Egfr*) involved in proliferation, (c) interleukin‐17F (*Il17f*), a pro‐inflammatory cytokine, (d) the lysine‐specific demethylase hairless (*Hr*), a histone demethylase, (e) deoxyribonuclease gamma (*Dnase1l3*), an enzyme with hydrolytic DNA activity. Nucleus associated upregulated transcripts are: (f) *Arnt2* coding for Aryl hydrocarbon receptor nuclear translocator 2, a transcription factor and (g) the protein FAM110C (*Fam110c*) known for interactions with microtubules and nucleus. Other upregulations are (h) phosphatidate phosphatase LPIN3 (*Lpin3*) involved in lipid synthesis, (i) monoacylglycerol lipase ABHD6 (*Abhd6*) forming intraluminal vesicles and (j) the glycosylating protein alpha‐2,8‐sialyltransferase 8F (*St8sia6*). Genes linked to immune functions are upregulated, including (k) the signaling factor SH2 domain‐containing protein 1B (*Sh2d1b*), (l) the tetraspanin leukocyte surface antigen CD53 (*CD53*), and (m) the viral response component 2'‐5'‐oligoadenylate synthase‐like protein 2 (*Oasl*).

## Discussion

### Optimum MTA addition levels during growth are 250–300 μm and stimulate cell cycle arrest, increase cell size and ensure high viability

We investigated concentration and time dependency of MTA addition in CHO cell cultures to elucidate the impact of these factors on the CSPs. Different effector levels at 48‐h cultivation time (Figs 1 and 2) revealed clear concentration dependency. Effects on growth, cell cycle and cell size were affected by different concentrations plateauing > 350 μm. CSP maxed out at about 250–300 μm. Furthermore, the two highest concentrations c3 and c4 even disclosed negative effects as decreasing viability and CSP. Noteworthy, effector levels < 350 μm ensured higher viabilities and higher CSPs than REF.

Similar dependencies of effector levels on CHO growth were observed for catechins trapping cells in S‐phase [48]. By analogy, treatment of cells with AMP is concentration‐dependent and resulted in S‐phase accumulation. As a consequence, CSP increased [49].

Further investigations on optimum MTA additions showed that supplementation during exponential growth is most beneficial (Fig. 3). Coinciding growth‐dependent cell size increase may further support the effect. Apparently, the combination of cell cycle arrest, high viability, and increasing cell size defines a key scenario for boosting CSP.

Many studies outlined the boosting effect of temporary cell cycle arrest on CSP although independent of a specific cell cycle phase [24, 28, 32, 41, 50, 51]. In several CHO cell lines, CSP and cell size correlated [28]. However, cell size increase is linked to cell cycle [25, 26, 27] which makes the independent study of each impact hardly possible. Consequently, the combinatorial benefit of cell cycle arrest with increased cell size, still ensuring high cell viability, should be concluded as beneficial for high CSP. Moreover, impaired cell growth yields less biomass formation and allows to use redundant energy and metabolic precursors for protein production [52]. Apparently, MTA initiates the beneficial combination when an optimum effector level of 250–300 μm is installed during exponential growth in the medium.

### Fundamental cell engineering strategies

Cell engineering for improved CSPs focus on engineering apoptosis, metabolism, cell cycle and protein secretion [53]. Transcript studies of low and high producers revealed that high recombinant protein formation negatively correlates with gene expression of cell cycle, metabolic RNA and protein processes [54]. Enhanced gene expression was observed in protein folding, cell survival, cell growth, vesicular trafficking and cytoskeleton organization [55]. As the map of functional gene annotations is still fragmented for CHO, identification of promising novel gene functions is necessary.

### The role of the cytoskeleton part actin for CSP after MTA addition

The water importer aquaporin 1 (encoded by *Aqp1*) was upregulated 84 h–96 h–144 h after MTA addition (Table 1) coinciding with increased cell size (Figs 2 and 3). This observation was seen in stress situations [56, 57] (e.g. hyperosmolarity) that caused increased intracellular protein content and CSP [58, 59]. In this experiment, the increased need of membrane molecules as glycerolipids could be satisfied by the upregulated phosphatide phosphatase LPIN3 (*Lpin3*) at 84 h–96 h. Co‐upregulation of *Lcp1* and *St14* occurred (84 h–96 h–144 h) coding for the actin‐associated enzyme plastin and matriptase [60, 61]. Noteworthy, actin microfilaments, microtubules and intermediate filaments compose the cytoskeleton which takes over crucial functions for cell shape, protein synthesis [62], transport [63] and secretion [64, 65]. Dinnis *et al*., [66] observed that actin, tubulin, or the actin‐binding cofilin demonstrated an important role in protein transport and secretion of high producers. Selection procedures for high producers revealed according to data with enhanced gene expression of actin‐related proteins [67]. Recently, Berger *et al*., [55] identified DEGs involved in cytoskeleton organization and vesicular trafficking as *Rassf9* that is linked to endosome recycling and is a trafficking regulator [68] in high producers. Our studies revealed upregulated genes (84 h–96 h) associated with intraluminal vesicles (monoacylglycerol lipase ABHD6 (*Abhd6*)) and protein processing (glycosylation) (alpha‐2,8‐sialyltransferase 8F (*St8sia6*)) in the Golgi (Table 1). Actin cooperates with polymerases via pre‐initiation complex influencing gene expression [69, 70] and is involved in cellular response to DNA damaging agents and toxins in CHO cells [71]. Next to the abovementioned actin‐related genes, *Add2* (beta‐adducin) at 96 h–144 h [72] and *Fscn1* (fascin) at 96 h [73] were upregulated in our data. Right after MTA addition (72 h–84 h) adenomatous polyposis coli protein 2 (*APC 2*), a transcription factor linked with actin [74] is downregulated. It is associated with microtubules and interphase [75] as protein FAM110C (*Fam110c*, upregulated at 84 h–96 h) that impairs cell cycle progression [76].

Several gene expressions related to cytoskeleton parts either for transport and secretion or cell growth are differentially regulated in our data highlighting their importance in the CSP enhancing mechanism of MTA.

### Genes encoding for cellular survival, transcriptional regulation and immune system

Plasminogen activator (*Plau*) upregulated at 84 h–96 h–144 h is a growth factor, mitogen and apoptotic reducer [77, 78]. Another upregulated gene (84 h–96 h) associated with cell growth, survival and transcription is the tumor‐suppressor ras association domain‐containing protein 6 (*Rassf6*) an important regulator of cell cycle arrest and apoptosis and whose upregulated family members were observed in high producers [55, 79]. The transcription factor aryl hydrocarbon receptor nuclear translocator 2 (*Arnt2)* correlated with cell proliferation [80] was downregulated at 72 h–84 h. At 84 h–96 h *Dnase1l3* and *Egfr* were upregulated encoding deoxyribonuclease gamma (Dnase1l3) and epidermal growth factor receptor (EGFR), respectively. Dnase1l3 is a apoptosis‐related factor [81] whereas EGFR is associated with DNA synthesis and proliferation [82]. *Atf5* (cyclic AMP‐dependent transcription factor ATF‐5) was upregulated at 84 h which agrees with studies searching for transcription and protein production regulators in CHO cells [83].

Next to growth and cellular survival factors, DEGs for histone proteins influenced transcription and replication [79]. Upregulation occurred for lysine‐specific demethylase hairless (*Hr*), a histone demethylase (84 h–96 h), that interacts with cell cycle regulation [84]. Additionally at 96 h–144 h, lysyl oxidase homolog 2 (*Loxl2*) and chromodomain‐helicase‐DNA‐binding protein 5 (*Chd5*), both histone modifying enzymes are upregulated [85, 86].

Genes involved in immune functions as SH2 domain‐containing protein 1B (*Sh2d1b*), CD53 (*CD53*), viral response component 2'‐5'‐oligoadenylate synthase‐like protein 2 (*Oasl*), interleukin‐17F (Il17f) (84 h–96 h) and interleukin‐11 (Il11) (72 h–84 h) were upregulated after MTA addition demonstrating a connection to the immune system and its connected signaling pathways [19].

DEGs regarding growth, survival and transcription including DNA modification point out the multi‐level effects of MTA that enhanced viability and CSP.

## Concluding remarks

MTA, the degradation product of SAM, boosts CSPs in an anti‐IL‐8‐producing CHO‐DP12, presumed that optimum MTA levels of 250–300 μm are installed for exponentially growing cells. Indeed, the rise of VCDs slowed down but CSPs increased up to +97%, even ensuring cell viabilities better than REF. Moreover, titers were comparable to REF in the best MTA addition case. These improvements coincided with cell cycle modulations, i.e. accumulations in S‐phase followed by elevated cell numbers in G2‐phase which both levelled out during cultivation. DEGs clearly showed upregulations of cytoskeleton, growth, survival and transcription‐associated genes as predominant regulation patterns. Although those DEGs may be qualified as a particular response on MTA next to its function as polyamine synthesis inhibitor that correlate with findings of other independent studies outlining that actin‐interacting proteins, cell proliferation and histone proteins are promising candidates for further cell engineering.

With MTA, a native compound is identified that clearly boosts CSPs after ‘simple’ medium addition. It is the key degradation product of SAM whose price will reduce with its microbial production [87]. MTA initiates regulation programs that deserve further investigations, not only because they may offer even further improvements but also because major findings may be translated to other production cell lines. Apparently, MTA addition positively stimulates cell cycle arrest, cytoskeleton and cell survival genes concomitantly, thereby addressing key topics of current cell line engineering. These findings should be considered for process intensification studies, especially for perfusion processes where improvements of CSPs are an important goal of optimization.

## Conflict of interest

The authors declare no conflict of interest.

## Author contributions

NV and RT designed the experiment. NV conducted the experiments and data analysis. JZ analyzed transcriptome data. NV and JZ interpreted transcriptome data. NV, JZ and RT wrote the manuscript.

## Supporting information


**Table S1.** List of differential expressed genes (FDR adjusted p‐values ≤ 0.05 and a log2‐fold‐change ≥|1|) between MTA treated cells and REF at different sampling points.

**Appendix S1.** Transcriptome analysis. Sample description, mapping statistics and counts.

## Data Availability

Processed transcriptome data is accessible in the supplemental part. Further data will be available from the corresponding author upon reasonable request.
