# Supplementary material for: Methylthioadenosine (MTA) boosts cell‐specific productivities of Chinese hamster ovary cultures: dosage effects on proliferation, cell cycle and gene expression
Source: FEBS Open Bio. 2020 Nov 11;10(12):2791–804. doi: 10.1002/2211-5463.13019 (PMC7714083; doi:10.1002/2211-5463.13019)
Supplement: Supplementary file 1 — Table S1. List of differential expressed genes (FDR adjusted p‐values ≤ 0.05 and a log2‐fold‐change ≥|1|) between MTA treated cells and REF at different sampling points. [file FEB4-10-2791-s001.pdf]

Supplemental Material 1

Methylthioadenosine (MTA) boosts cell specific productivities of Chinese hamster ovary cultures: dosage effects on proliferation, cell cycle and gene expression

Table S1. List of differential expressed genes (FDR adjusted p-values ≤0.05 and a log_2-fold-change ≥|1|) between MTA treated cells and REF at different sampling points.

Cultivation time	Gene name	Encoded protein	log_2FC	p_{adj}
60 h	LOC100758641	Unknown loci	-1.5462	2.09E-11
72 h	Apc2	Adenomatous polyposis coli protein 2	-2.0147	2.05E-05
	Il11	Interleukin-11	1.7590	9.04E-03
	LOC100752010	Unknown loci	-1.8965	3.28E-44
	LOC100752547	Unknown loci	-1.7611	6.47E-61
	LOC100752904	Unknown loci	-2.8233	6.96E-52
	LOC100754104	Unknown loci	-2.1791	1.22E-03
	LOC100757772	Unknown loci	-2.0797	4.80E-03
	LOC100758641	Unknown loci	-2.2051	3.56E-57
	LOC100759078	Unknown loci	2.6407	8.35E-06
	LOC100763833	Unknown loci	1.3991	1.06E-22
	LOC107979446	Unknown loci	-1.5823	2.07E-04
	LOC113834379	Unknown loci	2.4872	2.37E-12
84 h	Abhd6	Monoacylglycerol lipase ABHD6	1.6150	3.39E-14
	Apc2	Adenomatous polyposis coli protein 2	-2.6685	1.19E-11
	Aqp1	Aquaporin-1	1.6270	4.92E-07
	Arnt2	Aryl hydrocarbon receptor nuclear translocator 2	2.0281	1.81E-03
	Atf5	Cyclic AMP-dependent transcription factor ATF-5	-1.3981	1.00E-11
	Cd53	Leukocyte surface antigen CD53	1.2726	1.66E-02
	Dnase1l3	Deoxyribonuclease gamma	1.7149	2.64E-06
	Egfr	Epidermal growth factor receptor	1.9483	4.93E-02
	Fam110c	Protein FAM110C	3.3351	3.89E-02
	Hr	Lysine-specific demethylase hairless	1.7904	3.55E-11

	Il11	Interleukin-11	1.6337	1.34E-02
	Il17f	Interleukin-17F	1.3734	2.01E-02
	Lcp1	Plastin-2	1.3012	1.63E-02
	LOC100752010	Unknown loci	-1.8313	5.22E-41
	LOC100752320	Unknown loci	2.4102	2.92E-03
	LOC100752547	Unknown loci	-1.8263	4.61E-72
	LOC100752904	Unknown loci	-2.7420	5.12E-50
	LOC100754451	Unknown loci	1.7129	6.84E-04
	LOC100757491	Unknown loci	-1.4832	3.13E-03
	LOC100757772	Unknown loci	-2.1447	9.61E-04
	LOC100758065	Unknown loci	-1.7437	6.51E-04
	LOC100758641	Unknown loci	-1.8581	5.61E-31
	LOC100759078	Unknown loci	3.2187	7.24E-13
	LOC100763833	Unknown loci	1.3429	1.39E-16
	LOC100768011	Unknown loci	2.1813	1.33E-03
	LOC100772574	Unknown loci	1.8754	7.70E-05
	LOC103159878	Unknown loci	1.6027	2.92E-03
	LOC103160179	Unknown loci	1.5442	7.70E-05
	LOC103163365	Unknown loci	1.6926	2.19E-06
	LOC107979406	Unknown loci	2.0241	1.36E-02
	LOC107979446	Unknown loci	-1.7874	1.07E-09
	LOC113830954	Unknown loci	-1.5141	2.98E-04
	LOC113831402	Unknown loci	-1.5335	2.21E-06
	LOC113834379	Unknown loci	3.3442	2.24E-26
	Lpin3	Phosphatidate phosphatase LPIN3	1.4621	1.52E-04
	Oasl	2'-5'-oligoadenylate synthase-like protein 2	1.6231	7.03E-08
	Osr1	Protein odd-skipped-related 1	1.6940	4.93E-02
	Plau	Urokinase-type plasminogen activator	1.6420	1.84E-06
	Rassf6	Ras association domain-containing protein 6	3.0480	3.42E-05
	Sh2d1b	SH2 domain-containing protein 1B	2.6888	2.47E-02
	St14	Suppressor of tumorigenicity 14 protein homolog	1.7150	2.25E-06

	St8sia6	Alpha-2,8-sialyltransferase 8F	2.5774	5.18E-02
**96 h**	37135	unclassified gene	1.6601	3.44E-05
	Abhd6	Monoacylglycerol lipase ABHD6	1.5517	6.14E-13
	Acsl6	Long-chain-fatty-acid--CoA ligase 6	1.4768	2.77E-02
	Add2	beta-adducin	1.9814	4.20E-63
	Adgrd1	Adhesion G-protein coupled receptor D1	1.9627	2.90E-09
	Akr1d1	Aldo-keto reductase family 1 member D1	3.0345	6.37E-07
	Aqp1	Aquaporin-1	1.9092	2.58E-27
	Arnt2	Aryl hydrocarbon receptor nuclear translocator 2	2.4951	1.73E-09
	Btbd16	BTB/POZ domain-containing protein 16	2.6161	1.53E-03
	Cacnb4	Voltage-dependent L-type calcium channel subunit beta-4	1.4605	6.18E-07
	Caps2	Calcyphosin-2	1.9403	2.23E-04
	Cd53	Leukocyte surface antigen CD53	1.7555	1.06E-27
	Cdkl4	Cyclin-dependent kinase-like 4	2.3503	1.41E-02
	Dnase1l3	Deoxyribonuclease gamma	1.9169	1.01E-10
	Egfr	Epidermal growth factor receptor	1.9510	2.04E-02
	Fam110c	Protein FAM110C	3.9865	1.10E-03
	Fbln5	Fibulin-5	2.3963	4.75E-08
	Fcer2	Low affinity immunoglobulin epsilon Fc receptor	2.3978	1.84E-02
	Fgf22	Fibroblast growth factor 22	-2.3868	5.67E-04
	Fscn1	Fascin	2.0475	5.63E-03
	Fubp1	Far upstream element-binding protein 1	-1.3968	3.20E-18
	Gda	Guanine deaminase	2.5229	7.09E-03
	Grap2	GRB2-related adaptor protein 2	3.1363	4.42E-04
	Hr	Lysine-specific demethylase hairless	2.2624	1.08E-28
	Il17f	Interleukin-17F	1.5799	9.27E-08
	Il7r	Interleukin-7 receptor	1.7915	1.08E-13
	Lama3	Laminin subunit alpha-3	1.8936	2.84E-02
	Lama4	Laminin subunit alpha-4	3.2260	2.04E-07
	Lcp1	Plastin-2	1.8680	3.54E-32
	LOC100752320	Unknown loci	2.6816	5.68E-05

	LOC100754451	Unknown loci	2.1444	7.02E-12
	LOC100758264	Unknown loci	2.6336	1.91E-07
	LOC100759078	Unknown loci	3.1636	2.42E-11
	LOC100761810	Unknown loci	1.8829	2.51E-05
	LOC100765213	Unknown loci	2.4075	8.59E-04
	LOC100767707	Unknown loci	1.6958	8.07E-04
	LOC100768011	Unknown loci	1.9758	1.21E-02
	LOC100768431	Unknown loci	2.0809	6.29E-06
	LOC100769250	Unknown loci	2.1826	8.94E-03
	LOC100771813	Unknown loci	1.5230	9.47E-03
	LOC100772574	Unknown loci	2.5184	1.49E-17
	LOC100773221	Unknown loci	3.1006	6.35E-05
	LOC103159534	Unknown loci	3.1549	4.40E-02
	LOC103159864	Unknown loci	1.7840	1.11E-09
	LOC103160097	Unknown loci	2.7651	1.37E-04
	LOC103160117	Unknown loci	1.8504	2.63E-08
	LOC103160179	Unknown loci	1.8616	5.64E-16
	LOC103161069	Unknown loci	1.9762	6.04E-06
	LOC103161216	Unknown loci	2.5399	5.21E-04
	LOC103161664	Unknown loci	1.5320	2.58E-14
	LOC103162509	Unknown loci	3.7144	5.03E-06
	LOC103163365	Unknown loci	1.5129	1.37E-02
	LOC103163978	Unknown loci	2.4338	1.28E-03
	LOC103164035	Unknown loci	1.8408	1.01E-03
	LOC103164463	Unknown loci	2.7724	7.76E-22
	LOC107977260	Unknown loci	1.5847	3.33E-02
	LOC107977336	Unknown loci	2.7754	5.18E-02
	LOC107977412	Unknown loci	1.8604	5.23E-04
	LOC107978123	Unknown loci	4.2850	8.60E-03
	LOC107979406	Unknown loci	2.1857	1.43E-03
	LOC107979531	Unknown loci	3.7050	2.20E-02

	LOC107979868	Unknown loci	1.9182	3.52E-07
	LOC107979936	Unknown loci	3.0577	3.81E-06
	LOC113830872	Unknown loci	1.7550	3.65E-03
	LOC113834379	Unknown loci	2.8868	3.86E-17
	LOC113834741	Unknown loci	1.6853	8.49E-04
	LOC113835325	Unknown loci	2.6836	1.54E-02
	Loxl2	Lysyl oxidase homolog 2	1.3176	2.60E-06
	Lpin3	Phosphatidate phosphatase LPIN3	1.5952	6.91E-09
	Mpeg1	Macrophage-expressed gene 1 protein	2.2254	8.00E-04
	Neurl3	E3 ubiquitin-protein ligase NEURL3	1.4171	1.25E-07
	Oasl	2'-5'-oligoadenylate synthase-like protein 2	1.5642	9.50E-07
	Olfml2b	Olfactomedin-like protein 2B	2.1188	6.12E-09
	Pglyrp2	N-acetylmuramoyl-L-alanine amidase	3.1533	2.50E-02
	Pik3r5	Phosphoinositide 3-kinase regulatory subunit 5	1.9506	3.16E-04
	Plau	Urokinase-type plasminogen activator	2.2809	2.16E-32
	Ppm1h	Protein phosphatase 1H	2.0878	8.26E-03
	Rassf6	Ras association domain-containing protein 6	2.8067	1.23E-04
	Sema4d	Semaphorin-4D	1.5042	1.25E-03
	Sema4f	Semaphorin-4F	3.2138	3.52E-05
	Sh2d1b	SH2 domain-containing protein 1B	2.8037	7.95E-03
	Slc17a6	Vesicular glutamate transporter 2	2.3757	6.18E-07
	Spn	Leukosialin	3.3465	1.82E-03
	Spp1	Osteopontin	1.9261	1.40E-04
	St14	Suppressor of tumorigenicity 14 protein homolog	2.2368	1.39E-22
	St8sia6	Alpha-2,8-sialyltransferase 8F	3.2315	6.91E-05
	Tcea2	Transcription elongation factor A protein 2	-1.7638	8.29E-03
	Tmprss11f	Transmembrane protease serine 11F	2.0498	2.38E-12
**144 h**	Add2	beta-adducin	1.6707	1.88E-07
	Adgrd1	Adhesion G-protein coupled receptor D1	1.9219	1.88E-11
	Akr1d1	Aldo-keto reductase family 1 member D1	3.6322	1.20E-05
	Aqp1	Aquaporin-1	2.0311	1.01E-48

	Chd5	Chromodomain-helicase-DNA-binding protein 5	1.4287	1.84E-02
	Dpysl3	Dihydropyrimidinase-related protein 3	3.5452	5.24E-03
	Fbn1	Fibrillin-1	2.1766	1.90E-02
	Il7r	Interleukin-7 receptor	1.7037	7.07E-05
	Lcp1	Plastin-2	1.7737	1.55E-25
	LOC100765213	Unknown loci	3.1663	5.46E-03
	LOC100769250	Unknown loci	2.9004	1.34E-07
	LOC100771813	Unknown loci	2.2455	1.39E-18
	LOC100771938	Unknown loci	3.9692	1.84E-02
	LOC100772574	Unknown loci	2.9161	2.07E-32
	LOC103160179	Unknown loci	1.8126	1.61E-13
	LOC103160858	Unknown loci	2.3540	4.56E-06
	LOC103161069	Unknown loci	2.2406	1.64E-11
	LOC103163104	Unknown loci	2.8482	4.73E-04
	LOC103163978	Unknown loci	2.6849	2.17E-04
	LOC103164463	Unknown loci	1.9957	5.22E-02
	LOC107977412	Unknown loci	1.7400	1.98E-02
	LOC107978048	Unknown loci	3.4223	1.48E-04
	LOC107978123	Unknown loci	4.2591	1.07E-03
	LOC107978153	Unknown loci	3.9118	1.32E-02
	LOC107979254	Unknown loci	2.9282	1.77E-03
	LOC107979493	Unknown loci	2.8049	2.84E-02
	LOC107979936	Unknown loci	2.4581	1.39E-04
	Loxl2	Lysyl oxidase homolog 2	1.3890	1.82E-11
	Mmp2	72 kDa type IV collagenase	2.3902	2.12E-04
	Mpeg1	Macrophage-expressed gene 1 protein	1.8577	6.15E-03
	Pdzrn3	E3 ubiquitin-protein ligase PDZRN3	2.0603	1.31E-02
	Pecam1	Platelet endothelial cell adhesion molecule	1.2802	3.92E-03
	Pglyrp2	N-acetylmuramoyl-L-alanine amidase	3.2871	1.98E-02
	Pik3cg	Phosphatidylinositol 4,5-bisphosphate 3-kinase catalytic subunit gamma isoform	1.7025	3.13E-02
	Plac1	Placenta-specific protein 1	1.4593	1.88E-07

	Plau	Urokinase-type plasminogen activator	2.1120	2.88E-20
	Sema4d	Semaphorin-4D	1.8857	6.55E-09
	St14	Suppressor of tumorigenicity 14 protein homolog	2.0575	1.45E-12
	Tmc1	Transmembrane channel-like protein 1	-1.9236	1.75E-03
	Tmprss11f	Transmembrane protease serine 11F	2.0424	3.24E-04
	Treml4	Trem-like transcript 4 protein	2.9737	3.23E-03
